# MRI characteristics of brain edema in preeclampsia/eclampsia patients with posterior reversible encephalopathy syndrome

**DOI:** 10.1186/s12884-021-04145-1

**Published:** 2021-10-03

**Authors:** Hui Mai, Zhiyu Liang, Zhanhang Chen, Zhaoran Liu, Yaxi Xu, Xuting Chen, Xiujian Du, Yuling Peng, Yonglu Chen, Tianfa Dong

**Affiliations:** 1 Department of Radiology, The Third Affiliated Hospital of Guangzhou Medical University, No. 63 Duobao Road, Guangzhou, 510150 China; 2 Department of Medical Imaging Center, Nanfang Hospital, Southern Medical University, No. 1838 Guangzhou Avenue North, Guangzhou, 510515 China

**Keywords:** eclampsia, preeclampsia, posterior reversible encephalopathy syndrome, brain edema, magnetic resonance imaging

## Abstract

**Background:**

The neuroimaging manifestations of eclampsia and preeclampsia often overlap, mainly presenting as posterior reversible encephalopathy syndrome (PRES). The purpose of this retrospective study was to compare the extent and nature of brain edema in eclampsia and preeclampsia patients with PRES based on MRI characteristics.

**Methods:**

One hundred fifty women diagnosed with preeclampsia-eclampsia and undergoing cranial MRI were enrolled; 24 of these were diagnosed as having eclampsia. According to clinicoradiologic diagnosis of PRES, eligible patients were classified as having eclampsia with PRES (group E-PRES) and preeclampsia with PRES (group P-PRES). A scale on T2W FLAIR-SPIR images was established to evaluate the extent of brain edema, and the score of brain edema (SBE) of both groups was compared. In patients of the two groups who also underwent DWI sequence, the presence or absence of hyperintensity on DWI and hypointensity on ADC maps were determined to compare the nature of brain edema. Furthermore, clinical and biochemical data of the two groups were compared.

**Results:**

The incidence of PRES in eclampsia patients was significantly higher than that in preeclampsia patients (87.50% vs. 46.03%, *P*<0.001). The SBE of all regions and typical regions in group E-PRES patients were significantly higher than those in group P-PRES patients (15.88±8.72 vs. 10.90±10.21, *P*=0.021; 8.52±3.87 vs. 5.01±4.19, *P*=0.002; respectively). The presence of hyperintensity on DWI was determined more frequently in group E-PRES patients than group P-PRES patients (71.43% vs. 32.00%, *P*=0.024). Age, systolic blood pressure, white blood cell count, neutrophil count and percentage of neutrophils were significantly different between the two groups (*P*<0.05).

**Conclusions:**

Certain MRI characteristics that reflect the extent and nature of brain edema were different between eclampsia and preeclampsia patients with PRES. Additional prospective studies are still required to explore whether these MRI characteristics of brain edema may further become a potential predictor for eclamptic seizures in preeclampsia patients with PRES.

## Background

Eclampsia is the most severe stage of the preeclampsia–eclampsia spectrum, with an incidence of approximately 3/1000 to 9/1000 in pregnant or recently delivered women [1, 2]. Despite significant improvement in medical care and the popularity of prenatal diagnosis in recent years, the worldwide incidence and mortality of eclampsia remain high, seriously threatening maternal and fetal lives. Therefore, the early prediction of eclampsia has always been one of the severe challenges in obstetrics [3].

Neuroimaging manifestations of eclampsia and preeclampsia often overlap, mainly presenting as posterior reversible encephalopathy syndrome (PRES) [4, 5]. PRES is a distinctive clinicoradiologic syndrome characterized by headaches, visual disturbances, and seizures with predominantly parieto-occipital vasogenic edema, occasionally with cytotoxic edema [4]. Thus far, the exact pathophysiological mechanism of preeclampsia-eclampsia with PRES has not been clarified and is still controversial [6–8]. Not much is known about whether differences exist in the extent and nature of brain edema between eclampsia and preeclampsia patients with PRES; moreover, a “threshold” trigger in the occurrence of eclampsia has not yet been verified [9, 10].

Cranial conventional magnetic resonance imaging (MRI) is the preferred imaging modality for preeclampsia-eclampsia patients with PRES [4]. Gao et al. used conventional MRI sequences to score the extent of brain edema in PRES patients and found that the score was significantly correlated with serum levels of lactate dehydrogenase [6]. Diffusion-weighted imaging (DWI) is another commonly performed MRI sequence and is highly sensitive for distinguishing between cytotoxic and vasogenic edema [4, 11]. Recent MRI studies have shown that DWI can reflect the changes of pathophysiology regarding PRES patients in the ictal or peri-ictal phase of epilepsy [9]. The purpose of this retrospective research was to compare the extent and nature of brain edema in eclampsia and preeclampsia patients with PRES based on the MRI characteristics.

## Methods

### Patients

The study protocol was approved by the institutional review board, and written informed consent was waived given the retrospective nature of the study.

In all, 150 women diagnosed with preeclampsia-eclampsia and undergoing cranial MRI from September 2012 to March 2020 were enrolled; 24 of these were diagnosed as having eclampsia. The inclusion criteria was as follows: (i) preeclampsia was diagnosed according to the diagnostic criteria established by the American College of Obstetricians and Gynecologists [12], and eclampsia was defined by new-onset seizures that could not be attributed to other causative conditions in these women; (ii) women with preeclampsia underwent cranial MRI within 3 days before or after the onset of symptoms such as headache, vomiting, visual disturbance, mental status changes, and consciousness impairment, while those with eclampsia underwent cranial MRI within 3 days before or after the onset of seizures; and (iii) patients without neurological diseases such as epilepsy, brain tumors, and congenital brain malformations, which were unrelated to preeclampsia-eclampsia. The exclusion criterion was as follows: (i) patients that lacked clinicoradiologic findings of PRES [4]; and (ii) patients with abnormal neuroimaging features unrelated to PRES [7]. Finally, women who met these criteria were classified as having eclampsia with PRES (group E-PRES) and preeclampsia with PRES (group P-PRES).

### MRI assessment

MRI were performed on a 3.0T scanner (Achieva, Philips Healthcare, Best, The Netherlands) with an 8-channel head coil.

Conventional MRI was performed for all study patients. The conventional MRI scan sequences and parameters were as follows: (i) axial T1-weighted (T1W) spin echo sequence (repetition time: 500 ms, echo time: 7.5 ms, number of signal averaged: 2, slice thickness: 6 mm, and matrix size: 328×196); (ii) axial T2-weighted (T2W) turbo spin echo sequence (repetition time: 5000 ms, echo time: 80 ms, number of signal averaged: 2, slice thickness: 6 mm, and matrix size: 328×196); and (iii) axial T2W fluid attenuated inversion recovery - spectral presaturation inversion recovery (FLAIR-SPIR) sequence (repetition time / inversion time: 7000/2200 ms, echo time: 156 ms, number of signal averaged: 2, slice thickness: 6 mm, and matrix size: 328×196). The imaging diagnosis of PRES was based on the MRI finding of brain edema, which presented as hypointense or isointense signals on T1W and as hyperintense signal on T2W and T2W FLAIR-SPIR [4]. The incidence of PRES in patients with eclampsia and preeclampsia was calculated and compared.

Amongst these conventional MRI sequences, T2W FLAIR-SPIR is considered to be the most sensitive scan technique for detecting brain edema lesions of PRES [4]; therefore, we established a scale for T2W FLAIR-SPIR images to score and evaluate the extent (distribution and degree) of brain edema. The distribution of brain edema was divided into 15 regions according to anatomy: (1, 2) bilateral occipital lobes; (3, 4) bilateral parietal lobes; (5, 6) bilateral frontal lobes; (7, 8) bilateral temporal lobes; (9, 10) bilateral insular lobes; (11, 12) bilateral basal ganglia, thalamus, internal capsule, and external capsule; (13) cerebellum; (14) brain stem; (15) corpus callosum. The degree of brain edema in each region was divided into 0–III grades: grade 0, normal signal, score: 0 point; grade I, single fleck, patch, or nodule of abnormal signal, score: 1 point; grade II, multiple flecks, patches, or nodules of abnormal signals without fusion, score: 2 points; grade III, large area fusion of abnormal signals, score: 3 points. The sum of scores in each region was the score of brain edema (SBE) of all regions. In addition, we defined bilateral occipital and parietal lobes as typical regions of PRES and the rest as atypical regions of PRES. We then evaluated the SBE of both groups for typical and atypical regions.

Furthermore, MRI DWI sequence images of the two groups were collected. Axial DWI with b values of 0 and 1000 s/mm^2^ utilized a single-shot echo planar imaging (repetition time: 2258 ms, echo time: 86 ms, number of signal averaged: 2, slice thickness: 6 mm, and matrix size: 152×122). An axial apparent diffusion coefficient (ADC) map was generated automatically on a voxel-by-voxel basis from the two b values. The presence or absence of hyperintensity on DWI with b value of 1000 s/mm^2^ and hypointensity on the ADC maps were determined to evaluate the nature of brain edema.

MRI assessment for each patient enrolled in this study was independently evaluated by two radiologists with 13 and 5 years of experience, respectively, blinded to the clinical and biochemical data. For quantitative variables, the average was used; for the cases with discrepancies in the assessment of qualitative variables, a consensus interpretation was reached by joint review.

### Clinical and biochemical data assessment

Clinical data of the two groups was collected, including age, primiparity, gravidity, parity, length of hospital stay, and blood pressure. The biochemical data of both groups were collected, including blood cell count and indicators of liver and renal function. Biochemical indicators and blood pressure were obtained immediately at the occurrence of seizures in group E-PRES patients and at the occurrence of symptoms in group P-PRES patients.

### Statistical analysis

Descriptive statistics for continuous variables were presented as the mean±SD, while categorical variables were presented as frequencies and percentages. We used independent sample *t*-test (normally distributed variables) or Mann-Whitney U-test (non-normally distributed variables) to compare the continuous variables of the two groups, and chi-square or Fisher’s exact test to compare the categorical variables. The intraclass correlation efficient (ICC) was used to evaluate the inter-observer repeatability of SBE measurement. An ICC value>0.75 indicates that the repeatability is excellent. All statistical analyses were performed using SPSS version 26.0 (IBM Corporation, Armonk, NY, USA). P<0.05 was considered to indicate statistical significance.

## Results

**Fig. 1:**
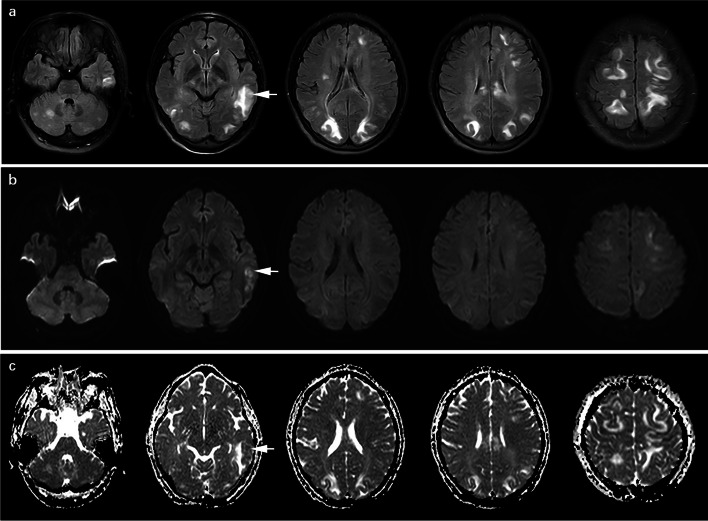
Cranial MRI of a 31-year-old woman with eclampsia with PRES: **a** axial T2W FLAIR-SPIR, **b** axial DWI with b value of 1000 s/mm^2^, **c** axial ADC map. SBE was evaluated according to the distribution and the degree of abnormal edema signal, which presented as heterogeneous hyperintense signal (arrow) on T2W FLAIR-SPIR (**a**). Large area fusions of abnormal edema signals were shown in corpus callosum, bilateral occipital, parietal, frontal, and temporal lobes; and multiple flecks, patches or nodules of abnormal edema signals without fusion were seen in the cerebellum and right basal ganglia. The SBE of typical regions, atypical regions, and all regions in this patient were 12, 19, and 31, respectively. Some of the abnormal edema signals in bilateral frontal lobes, left parietal and temporal lobes presented as hyperintense signals (arrow) on DWI (**b**), with the lesion in left temporal lobe locally appearing as hypointense signal (arrow) on ADC map (**c**)

**Fig. 2:**
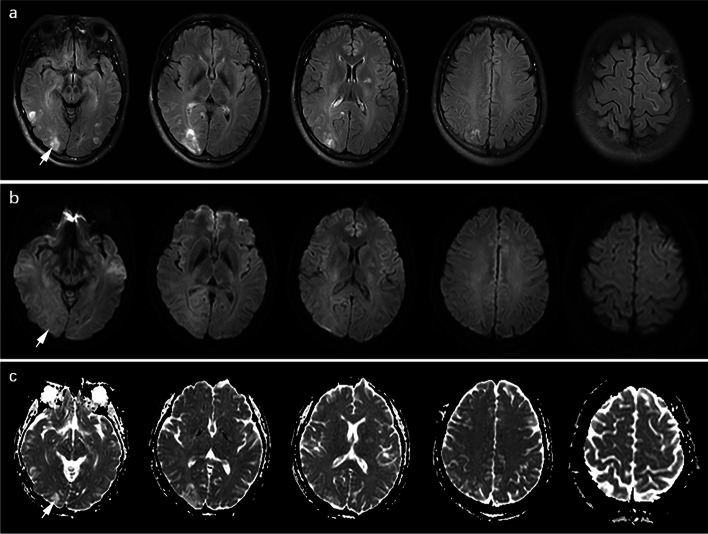
Cranial MRI of a 31-year-old woman with preeclampsia with PRES: **a** axial T2W FLAIR-SPIR, **b** axial DWI with b value of 1000 s/mm^2^, and **c** axial ADC map. SBE was evaluated according to the distribution and degree of abnormal edema signal, which presented as heterogeneous hyperintense signals (arrow) on T2W FLAIR-SPIR (**a**). Large area fusions of abnormal edema signals were shown in the right occipital and temporal lobes; and multiple flecks, patches, or nodules of abnormal signals without fusion were seen in the left basal ganglia, right parietal lobe, left occipital, temporal and frontal lobes. The SBE of typical regions, atypical regions, and all regions in this patient were 7, 9, and 16, respectively. The abnormal edema signals presented as isointense signals (arrow) on DWI (**b**) and as hyperintense signals (arrow) on ADC map (**c**)

In our study, PRES occurred in 87.50% eclampsia patients (21/24) and 46.03% preeclampsia patients (58/126). The incidence of PRES in eclampsia patients was significantly higher than that in preeclampsia patients (*P*<0.001). The inter-observer repeatability of SBE measurement indicated excellent with ICC>0.75. The SBE of all regions and typical regions in group E-PRES patients were significantly higher than those in group P-PRES patients (15.88±8.72 vs. 10.90±10.21, *P*=0.021; 8.52±3.87 vs. 5.01±4.19, *P*=0.002; respectively). The SBE of atypical regions in group E-PRES patients was slightly higher than that in group P-PRES patients, but this difference was not statistically significant (7.36±5.62 vs. 5.91±6.79, *P*=0.097). Table 1 shows the SBE of the two groups, and examples are shown in Figs. 1 and 2.

**Table 1:** SBE in eclampsia patients with PRES (group E-PRES) and preeclampsia patients with PRES (group P-PRES)

Parameter	Group E-PRES (*n*=21)	Group P-PRES (*n*=58)	T value	*P*
SBE of all regions	15.88±8.72	10.90±10.21	402.00	0.021
SBE of typical regions	8.52±3.87	5.01±4.19	377.50	0.002
SBE of atypical regions	7.36±5.62	5.91±6.79	460.00	0.097

*SBE* Score of brain edema, *PRES* Posterior reversible encephalopathy syndrome

14 group E-PRES and 25 group P-PRES patients were imaged using the MRI DWI sequence. The majority of brain edema lesions appeared as hyperintense or isointense signals on DWI and as hyperintense signals on the ADC map. Furthermore, coexistence of different signals on DWI and ADC maps of the affected areas was sometimes observed. The presence of hyperintensity on DWI was determined in 71.43% group E-PRES patients (10/14) and 32.00% group P-PRES patients (8/25), with statistically significant differences (*P*=0.024). The hypointense signal on ADC map presented in group E-PRES patients (4/14, 28.57%) was slightly more frequent than that presented in group P-PRES patients (2/25, 8.00%), but this difference was not statistically significant (*P*=0.163). Table 2 shows the features of DWI and ADC map on brain edema lesions in the two groups, and examples are shown in Figs 1 and 2.

**Table 2:** The features of DWI and ADC map on brain edema lesions in eclampsia patients with PRES (group E-PRES) and preeclampsia patients with PRES (group P-PRES)

Parameter	Group E-PRES (*n*=14)	Group P-PRES (*n*=25)	*P*
Hyperintensity on DWI			0.024
Present	10 (71.43%)	8 (32.00%)	
Absent	4 (28.57%)	17 (68.00%)	
Hypointensity on ADC map			0.163
Present	4 (28.57%)	2 (8.00%)	
Absent	10 (71.43%)	23 (92.00%)	

*DWI* Diffusion weighted imaging, *ADC* Apparent diffusion coefficient, *PRES* Posterior reversible encephalopathy syndrome

Group E-PRES patients were significantly younger than group P-PRES patients (26.24±6.65 vs. 33.41±4.42 years old, *P*<0.001). Systolic blood pressure, white blood cell count, neutrophil count, and percentage of neutrophils in group E-PRES patients were significantly higher than those in group P-PRES patients (*P*<0.05). Other clinical and biochemical parameters collected in this study did not significantly differ between the two groups. Table 3 shows the clinical and biochemical parameters of the two study groups.

**Table 3:** Clinical and biochemical parameters in eclampsia patients with PRES (group E-PRES) and preeclampsia patients with PRES (group P-PRES)

Parameter	Group E-PRES (*n*=21)	Group P-PRES (*n*=58)	T or Χ^2^ value	*P*
Age (years)	26.24±6.65	33.41±4.42	248.00	<0.001
Primiparity	15 (71.43%)	29 (50.00%)	2.87	0.090
Gravidity (No.)	2.19±1.08	2.97±1.90	475.00	0.128
Parity (No.)	1.38±0.67	1.74±1.02	480.50	0.110
Length of hospital stay (day)	9.10±3.30	12.07±22.11	553.50	0.534
Systolic blood pressure (mmHg)	173.19±17.14	183.78±18.61	388.50	0.014
Diastolic blood pressure (mmHg)	110.95±13.97	114.81±10.34	1.33	0.188
White blood cell count (10^9^/L)	15.39±5.39	11.73±3.31	346.00	0.004
Neutrophil count (10^9^/L)	10.04±5.49	9.45±3.32	363.00	0.006
Percentage of neutrophils (%)	83.18±10.67	78.63±9.01	423.50	0.040
Platelet (10^9^/L)	193.86±100.30	184.81±86.43	597.50	0.898
Alanine transaminase (U/L)	54.58±117.31	32.36±54.47	504.00	0.244
Aspartate aminotransferase (U/L)	79.17±171.84	60.83±136.78	493.50	0.200
Uric acid (umol/L)	526.87±145.96	475.25±96.03	-1.51	0.144
Serum creatinine (umol/L)	66.24±14.63	71.71±26.47	575.50	0.710

*PRES* Posterior reversible encephalopathy syndrome

## Discussion

Previous studies have shown that abnormal imaging findings of PRES typically associated with eclampsia were also observed frequently in preeclampsia patients without seizures [5, 8, 13]. In this study, although the incidence of PRES in eclampsia patients was significantly higher than that in preeclampsia patients, nearly half the preeclampsia patients with symptoms also experienced PRES. Fang et al. further reported that there were no significant differences between preeclampsia and eclampsia patients in the distribution score or degree score of brain edema on MRI [10]. The sample size of our study was similar to that of Fang et al’s study. However, our study found that the SBE of typical regions, atypical regions, and all regions in group E-PRES patients were all higher than those in group P-PRES patients; furthermore, the SBE of typical regions and all regions were significantly different between the two groups. The distribution score and degree score of brain edema were combined into the measure of SBE in our study rather than counted independently as in Fang et al’s study, which may be a more comprehensive scale for assessing the extent of brain edema. This scoring method for brain edema has been rarely mentioned in the literature except for in a previous study, which found that this scoring method for brain edema showed positive correlation with the ratio of soluble endoglin to placental growth factor that reflected the degree of vascular endothelial injury in preeclampsia patients with PRES [14].

The results showed that the difference in SBE of all regions between the two study groups was mainly due to the difference in the edema score of typical regions. The preferential distribution of posterior parietal and occipital lobe regions in PRES is not well understood. It is generally accepted that posterior circulation arteries have sparser mural sympathetic innervation and are thinner than other cerebral arteries, which make them more vulnerable to endothelial dysfunction and more prone to autoregulation breakthrough in acute hypertension [7, 15]. Interestingly, in our study, the systolic blood pressure of group E-PRES patients was not higher, rather significantly lower than that of group P-PRES patients. Previous studies have suggested that the severity of hypertension did not seem to be consistent with the onset of eclampsia [16]. In the pathogenesis of eclamptic seizures, hypertension alone is not the only trigger; endothelial dysfunction might be a more important contributing factor [8, 17, 18].

Endothelial dysfunction is often positively associated with the degree of inflammation [10, 19]. In our study, the inflammatory indexes in group E-PRES patients were significantly higher than those in group P-PRES patients, which was similar to the findings of previous research [13]. In a rat eclampsia-like model, biochemical evidence suggesting that systemic inflammation might lower the threshold of seizures has been reported [20]. In addition, Li et al. indicated that magnesium sulfate reduced neuroinflammation and brain edema to prevent eclampsia-like seizures [21].

The majority of lesions with hyperintense signals on T2W FLAIR-SPIR appear as hyperintense or isointense signals on DWI with increased ADC values typically consistent with mobile water in the involved vasogenic edema in PRES. Nonetheless, our study found that the presence of hyperintensity on DWI was determined more frequently in group E-PRES than in group P-PRES patients, and hypointensity on the ADC map presented in group E-PRES patients were slightly more frequent than that presented in group P-PRES patients. Many studies have shown that hyperintensity of DWI with pseudo-normalized ADC values seems to imply the earliest sign indicating the progression of severe vasogenic edema to cytotoxic edema; nevertheless, hyperintensity of DWI with decreased ADC values represents cytotoxic edema and seems to be correlated with nonreversible cerebral infarction [7, 22]. Thus, our study and previous MRI studies have shown that DWI sequence can evaluate the nature of brain edema by reflecting the diffusion motion of cerebral water molecules, which might reveal the changes of pathophysiology in the peri-ictal phase of eclampsia patients with PRES [9, 23].

Group E-PRES patients were significantly younger than group P-PRES patients, which was consistent with other studies [10, 24, 25]. Low maternal age was considered as a risk factor for developing eclampsia, which might be related to other factors including primiparity, a lack of education and prenatal care, among others [10, 24, 25].

Our study has some limitations. First, given the retrospective nature of the study, inherent variations and biases of the study design need to be acknowledged; the data were collected from medical records, and the results might be affected by the completeness and accuracy of these records; furthermore, this was a single-center study with relatively few cases. Therefore, further prospective multi-center studies with more cases are expected. Second, DWI sequences of some patients in the two groups were not available, so the nature of brain edema in these patients could not be determined. Third, most patients did not receive follow-up MRI examination; hence, subsequent changes of brain edema could not be tracked and evaluated.

## Conclusions

What triggers the occurrence of eclampsia in preeclampsia patients with PRES is still unknown. This retrospective study has suggested that certain MRI characteristics that reflect the extent and nature of brain edema are different in eclampsia and preeclampsia patients with PRES. However, additional prospective studies are still required to explore whether these MRI characteristics of brain edema may further become a potential predictor for eclamptic seizures in preeclampsia patients with PRES. Given the high mortality related to intracranial complications in eclampsia patients, we recommend early consideration of cranial MRI in the management of preeclampsia patients to evaluate the presence or absence of PRES as well as the extent and nature of brain edema.

## Data Availability

The datasets used and analyzed during the current study are available from the corresponding author on reasonable request.

## Abbreviations

PRES: Posterior reversible encephalopathy syndrome
MRI: Magnetic resonance imaging
DWI: Diffusion-weighted imaging
T1W: T1-weighted
T2W: T2-weighted
FLAIR: Fluid attenuated inversion recovery
SPIR: Spectral presaturation inversion recovery
SBE: Score of brain edema
ADC: Apparent diffusion coefficient
ICC: Intraclass correlation efficient
